# Insecticidal Activity of Some Major Essential Oil Components against *Metopolophium dirhodum* and Its Predators

**DOI:** 10.3390/plants13131863

**Published:** 2024-07-05

**Authors:** Roman Pavela, Matěj Novák

**Affiliations:** 1 Crop Research Institute, 161 06 Prague, Czech Republic; pavela@vurv.cz; 2 Department of Plant Protection, Czech University of Life Sciences Prague, 165 00 Prague, Czech Republic; 3 Department of Plant Biotechnology, College of Life Sciences and Biotechnology, Korea University, Seoul 02841, Republic of Korea

**Keywords:** terpenoids, binary mixture, botanical pesticides, essential oils, synergism aphids, *Aphidoletes aphidimyza*, Chrysoperla carnea

## Abstract

Essential oils (EOs) are plant metabolites with important insecticidal effects. Nevertheless, information on the efficacy of the major substances on aphids and their natural enemies is still missing. The objective of this paper is, therefore, to identify the efficacy of selected EO majority substances—β-citronellol, carvacrol, isoeugenol, and linalool, including their binary mixtures—on the mortality and fertility of the aphid *Metopolophium dirhodum*, an important cereal pest. The best efficacy was proven for the binary mixture of β-citronellol and linalool (1:1 ratio), for which the estimated LC_50(90)_ is 0.56(1.58) mL L^−1^. This binary mixture applied in sublethal concentrations significantly reduced aphid fertility. It was found that the phenomenon can be attributed to β-citronellol, as the females treated with LC_30_ laid 45.9% fewer nymphs, on average, compared to the control. Although β-citronellol and linalool, including their 1:1 mixture, showed very good efficacy on aphid mortality, they were, on the other hand, very friendly to the larvae of *Aphidoletes aphidimyza* and *Chrysoperla carnea*, which are important aphid predators. Based on our results, the newly discovered synergically acting binary mixture β-citronellol/linalool can be recommended as an efficient substance suitable for the further development of botanical insecticides used against aphids.

## 1. Introduction

Essential oils (EOs) are complex mixtures of volatile compounds, including primarily oxygenated and non-oxygenated hydrocarbons, that are most commonly monoterpenes (representing 80% of EO composition) and sesquiterpenes [1,2]. EOs are most commonly composed of 20 to 60 substances, of which 1–3 typically comprise the majority proportion, frequently representing up to 90% of the overall EO composition [3,4,5]. EOs are carriers of the fragrance and flavor of aromatic plants, which have traditionally been used in food processing, where either the plants themselves or extracts or EOs obtained from them are used as seasoning ingredients [5,6,7]. They are also valued in medicine and cosmetics, which make use of their healing properties, including the positive effect of fragrances on the human psyche [6]. EOs are one of the means of communication for plant–insect interaction. Plants synthesize EOs, for example, to better attract pollinators or, conversely, to discourage pests [8]. They can thus be considered one of the substances directly connected to the natural defensive mechanisms of plants. As a result, they have multiple pesticidal properties, which have come to the foreground of intensive research in recent decades. The reason for this is that EOs can be used successfully as active substances in botanical pesticides, including insecticides [7,9]. EOs appear to be highly suitable for the development of botanical insecticides [7], as they show very promising acute and chronic toxicity against numerous vectors and plant pests, including aphids [10,11,12], while being very environmentally friendly, as they undergo relatively fast natural biodegradation and are tolerant to many non-target organisms [13]. It can rightly be assumed that their potential residues have no negative effects on human health since they are commonly found in our food, whereas, in contrast, positive impacts on human health are attributed to EOs [14]. Moreover, the individual substances contained in EOs demonstrate several mechanisms of action so that their combination significantly reduces the risk of pathogens and pests developing resistance [9].

On the other hand, using EOs as active substances within botanical insecticides has a number of limiting factors that also prevent their broader application in practice. Namely, EO content in plant biomass is relatively low and typically represents (0.1)0.5–1.5(5)% of plant dry matter [9], which means that the amount of EOs acquired annually is very limited, and manufacturers face a risk of a lack of material for botanical insecticides. Moreover, the level of active substances in EOs can differ depending on climate and soil conditions and biomass harvesting and processing methods [9]. Another limiting factor is the relatively low persistence of the effect due to the volatility and environmental biodegradation of EOs. Whereas the persistence of the effect or even the insecticidal efficacy of EOs can be resolved by appropriate formulation methods, such as encapsulation and micro/nano-formulations [15,16], the quantity of EOs acquired annually for the manufacturing of botanical insecticides can be increased by the development of novel cultivation methods [9], which are nonetheless still limited by the amount of biomass obtained.

For these reasons, it is justified to assume that it is more appropriate to use the major substances in EOs for the development of botanical insecticides, where they can be obtained synthetically in large quantities and where they can be expected to display all the positive properties of natural EOs as described above. At the same time, however, it is important to have sufficient relevant information about not only the effects of the individual terpenes on target organisms but also their impact on non-target organisms, most importantly predators and parasitoids. Moreover, there is very little information about the lethal and sublethal doses of terpenes on pests, although it can be assumed that even sublethal concentrations may have a negative effect on insect fertility and fecundity [17,18].

The objective of this paper was, therefore, to evaluate the efficacy of four major substances in EOs—carvacrol, β-citronellol, isoeugenol, and linalool—which show very promising insecticidal efficacy [19,20,21,22] while also being industrially available substances against *Metopolophium dirhodum* Walker (Hemiptera: Aphididae) aphids, an important cereal pest in Europe. The aphid sucks the sap from the plant, which leads to a reduction in the quality of the grain [23]. In addition, *M. dirhodum* is an important vector of barley yellow dwarf virus (BYDW), which causes symptoms such as yellowing, reddening, leaf curling, leaf rolling, and reduction in size, number of ears, and grains [24]. Protection against *M. dirhodum* includes the use of synthetic insecticides, mainly pyrethroids, organophosphates, and neonicotinoids, but the frequent use of synthetic insecticides, as with other pests, leads to the emergence of resistant populations. For example, some populations of *M. dirhodum* have been found to be resistant to the insecticides thiamethoxam, imidacloprid, abamectin, and omethoate [25]. In addition, many of these insecticides are toxic to non-target insects.

For these reasons, it is necessary to search for new active substances with new mechanisms of action (MoAs). At the same time, they must be safe for non-target organisms. We identified not only the effect of selected substances on the acute toxicity of aphids, but we also clarified the synergic relationships of their binary mixtures, which is an important precondition for the development of new unique combinations of active substances with different mechanisms of action [10,26,27]. In addition, we identified for the most effective binary mixture the effect of application to two non-target organisms—*Aphidoletes aphidimyza* Rondani (Diptera: Cecidomyiidae), one of the most important aphid predators used in biological plant protection [28], and *Chrysoperla carnea* Stephens (Neuroptera: Chrysopidae), an important predator of sap-sucking insects [29]. To the best of our knowledge, this is the first paper to make such a comprehensive assessment of the efficacy of carvacrol, β-citronellol, isoeugenol, and linalool on the life cycle and developmental characteristics of aphids and their natural enemies.

## 2. Results

### 2.1. Acute Toxicity against M. dirhodum

Although all four tested substances showed a very good contact efficacy on the mortality of adults of *M. dirhodum*, no significant differences between the substances were identified (Table 1). The significantly most effective was β-citronellol, for which, admittedly, the LC_50_ was estimated (0.59 mL L^−1^) at approximately the same level as for carvacrol (0.60 mL L^−1^), although the applicable values of LC_90_, which can be regarded as more important from the point of view of practice, differed substantially (1.78 and 2.82 mL L^−1^ for β-citronellol and carvacrol, respectively). Linalool was the least effective, for which the LC_50(90)_ was estimated at 3.68(9.46) mL L^−1^.

### 2.2. Acute Effects of the Binary Mixtures against M. dirhodum

Although the highest lethal concentration was estimated for linalool, it showed, on the other hand, the greatest synergic effect in a binary mixture with β-citronellol (Table 2), with an identified mortality of 83.8% rather than the expected 40.4%. Carvacrol also showed a very promising synergic effect in binary mixtures with linalool and isoeugenol.

Since the binary mixture combination of linalool and β-citronellol showed the best synergic effect, this combination was selected for our further research. It was first experimentally proven that the mixing ratio of 1:1 is an optimal synergic mixture (Table 3), as it was precisely this ratio that showed significantly the highest mortality rates of 83.5 and 97.8% when applied at concentrations of 1.5 and 2.5 mL L^−1^, respectively.

For the binary mixture linalool/β-citronellol (1:1), the LC_50(90)_ for *M. dirhodum* was estimated at 0.56(1.58) mL L^−1^ (Table 4).

As part of the study of the effects of lethal and sublethal concentrations on aphid fertility, we applied concentrations corresponding to LC_30_ and LC_50_ for both linalool and β-citronellol alone and for their binary mixture (1:1). It was found that only β-citronellol significantly reduced the fertility of surviving females (Table 5), as females treated with LC_30_ laid 45.9% fewer nymphs, on average, compared to the control. This phenomenon also occurred for the mixture, but only in females treated with LC_50_. Thus, overall, the potential natality decreased by more than 60% for both β-citronellol and its mixture with linalool.

### 2.3. Acute Toxicity against Aphidoletes aphidimyza and Chrysoperla carnea

Although β-citronellol and linalool, including their 1:1 mixture, showed very good efficacy on aphid mortality, they were, on the other hand, very friendly to the tested non-target organisms. After application of a concentration corresponding to LC_90_, under identical application and post-application conditions, we detected mortality rates lower than 5% for *A. aphidimyza* larvae (Table 6). For *C. carnea* larvae (Table 7), we observed significantly higher mortality compared to the control only for β-citronellol (35.3%), but the 1:1 mixture showed lower mortality (19.6%), which did not differ significantly from the negative control (9.0%), whereas the positive control in the form of Neudosan, a product registered against aphids, caused a fatal 100.0% mortality among *C. carnea* larvae.

## 3. Discussion

Essential oils (EOs) show significant pesticidal effects, including insecticidal ones, and they have become the focus of research in recent decades, aiming to find EOs that could be used as suitable active ingredients of botanical insecticides. Although the insecticidal efficacy of many EOs is known, there is very little information on the efficacy of their individual major compounds against aphids. This is despite the fact that aphids are among the most common pests of many cultivated crops, and they are becoming resistant to traditional active substances such as pyrethroids, neonicotinoids, and carbanoids [25]. In this regard, we evaluated the insecticidal potential of four major components of EOs.

Based on our preliminary tests and the results of other authors [11,19,20,21,22], we chose four substances that had shown promising aphicidal results in our previous tests and which comprise the major substances in EOs from numerous plants. For example, β-citronellol is a major component of the EOs of *Eucalyptus citriodora* (Hook.) K.D. Hill & L.A. Johnson Bor [30], *Pelargonium roseum* (Andrews) DC [31], as well as *Rosa* × *damascena* Mill [32]. β-Citronellol is used in industries such as perfumes, beverages, food, and pharmaceuticals [33]. Carvacrol is a major component in the EOs of *Origanum vulgare* L., *Lippia origanoides* Kunth., *Origanum majorana* L., *Origanum compactum* Benth. *Satureja hortensis* L., *Satureja thymbra* L., *Satureja wiedemanniana* (Avé-Lall.) Velen., *Thymbra sintenisii* Bornm. & Azn., *Thymbra spicata* L., *Thymus capitatus* (L.) Hoffsgg. & Link, etc. [9], and *Thymus vulgaris* L. [34]. Carvacrol is used as a disinfectant, fungicide, and fragrance ingredient in cosmetic formulations [35]. Isoeugenol is present naturally, for example, in the EOs of *Myristica fragrans* Houtt. [36], *Cananga odorata* (Lam.) Hook. f. & T. Thomson [37], *Eugenia caryophyllata* Thunb. [38] and *Ocimum basilicum* L. [39]. Isoegenol is used in the perfume and cosmetics industries [40]. Linalool is a major component, e.g., in the EOs of *Cinnamomum camphora* (L.) J. Presl, *Ocimum basilicum* L., *Coriandrum sativum* L., and *Lavandula angustifolia* Mill. [9]. Linalool is a key compound in the manufacture of a wide range of domestic products, cosmetics, and fragrance chemicals such as geraniol, nerol, citral, and its derivatives. It is also a key compound in the synthesis of vitamins A and E in the pharmaceutical industry [41].

This paper finds that all the tested substances showed a promising efficacy against adults of *M. dirhodum*. The most effective substances are β-citronellol and carvacrol, for which the LC_50_ was estimated at approximately 0.6 mL L^−1^. That is much less compared to some other EOs that other authors have considered to be promising active substances suitable for the development of botanical insecticides. For example, for the EOs produced from the leaves of *Eucalyptus citriodor*, in which β-citronellol is one of the majority components, the LC_50_ and LC_90_ for *Myzus persicae* were estimated at 4 mL L^−1^ and 11.5 mL L^−1^, respectively [30]. For the EOs of *Satureja intermedia* C. A. Mey., in which carvacrol is one of the majority components, the LC_90_ for *Aphis nerii* was estimated at 1.37 mL L^−1^ [42]. Linalool was the least effective, for which the LC_50_ was estimated at 3.68 mL L^−1^. In spite of that, the substance can still be considered more effective than, for example, the EOs of *Lavandula angustifolia* [12], which contain a majority proportion of this substance and for which the LC_50_ was estimated at 20 mL L^−1^. On the other hand, it is important to bear in mind that it is very difficult to compare our results with those of others because the efficiency of EOs may be influenced by different methodologies, different aphid sizes, and different application methods and post-application conditions, particularly temperature [43]. Likewise, antagonistic or synergic relationships between majority substances may influence the final efficacy of EOs or binary mixtures [10,26].

The study of synergic and antagonistic relationships is very important in the development of new botanical insecticides based on EOs or mixtures of their majority active substances. A suitable combination of binary or complex mixtures may not only increase the efficiency [10,26] but it can also be assumed that synergic mixtures show several different mechanisms of effect, which prevent their rapid detoxication by the insect and thus prevent the development of resistant insect populations [27,44]. The present paper is the first to describe the synergic relationships between β-citronellol and linalool as part of their aphicidal efficacy. It had been identified before, however, that these substances in a binary mixture show a significant synergic effect against larvae of *Spodoptera littoralis* (Boisduval, 1833) and *Culex quinquefasciatus* Say, 1823, and a synergic effect had been found between linalool and isoeugenol [10]. Our work is thus in accordance with prior observations.

Information about the effects of lethal and sublethal doses and concentrations is equally important. This is because we can assume that the practical blanket application of insecticides may not be even, and parts of the crop can, therefore, be treated with lethal or even sublethal concentrations. It had previously been found that lethal and sublethal concentrations or doses of EOs may reduce insect fertility and vitality [45,46]. Our paper is the first to describe that this may not always be the case because while β-citronellol significantly reduced the fertility of surviving females, with females treated with LC_30_ laying 45.9% fewer nymphs on average compared to the control, no such phenomenon was identified for linalool. The binary mixture β-citronellol/linalool (1:1) was also found to reduce fertility and the associated potential natality of aphids. Reducing the number of aphids in cereal crops is very important because it significantly reduces the damage caused by their sap-sucking. Moreover, weakened colonies can be expected to be attacked and destroyed by their natural enemies at a faster pace [47]. It is, therefore, important that insecticides, including botanical insecticides, be friendly to non-target organisms. The present paper finds that the tested 1:1 binary mixture was friendly to both *A. aphidimyza* larvae and *C. carnea* larvae. The observed mortality rates did not differ significantly from the negative control, while the positive control in the form of Neudosan (active ingredient: potassium salts of fatty acids), which is registered against aphids, caused a fatal 100.0% mortality of *C. carnea* larvae. We are aware, however, that further tests that would clarify the effect on non-target organisms will be necessary. It will be equally important to find out the efficacy on other aphid species or other pests, not only in cereal crops. An integral component of further research should be formulation methods [15] that could increase or extend the efficacy, which will have to be tested in field experiments.

In conclusion, based on our results, we can recommend the mixture of β-citronellol/linalool at a mixing ratio of 1:1 (*v*:*v*) as a synergic active substance suitable for the further development of botanical insecticides used against aphids. The mixture shows a very promising aphicidal efficacy (LC_90_ = 1.6 ml L^−1^), its sublethal concentrations reduce the fertility of *M. dirhodum*, and it is friendly to non-target organisms, such as *A. aphidimyza* and *C. carnea* larvae.

## 4. Materials and Methods

### 4.1. Compounds

Isoeugenol (analytical standard), carvacrol (natural, purity 99%, FoodGrade), (±)-β-citronellol (Techn., purity 90–95%, for gas chromatography), and (-)-linalool (purity > 95.0%) were obtained from SigmaAldrich (Prague, Czech Republic). The substances were emulsified using Tween 20 (SigmaAldrich, Prague, Czech Republic). The positive control consisted of Neudosan (Neudorff W. GmbH. KG, Germany, active substance: potassium salts of fatty acids 515 g kg^−1^).

### 4.2. Insects

*Metopolophium dirhodum* adults (wingless females, 1–2 days old) were obtained from laboratory mass-rearing (Crop Research Institute, Prague, Czech Republic). Colonies of *M. dirhodum* aphids were maintained for >20 generations on wheat plants (*Triticum aestivum* L.) in insect cages of dimensions 35 × cm 35 cm × 60 cm at a temperature of 21 ± 3 °C, 65 ± 5% R.H., and a 16:8 (L:D) photoperiod.

Third instar larvae of *Aphidoletes aphidimyza* were obtained from established laboratory breeding (Crop Research Institute, Prague, Czech Republic). The insects were reared in insect cages of dimensions 35 cm × 35 cm × 60 cm and were fed on *Myzus persicae* (Sulzer) aphids that were on *Brassica oleracea* var. gongylodes L. Breeding was maintained at a temperature of 21 ± 3 °C, 65 ± 5% R.H. and a 16:8 (L:D) photoperiod.

*Chrysoperla carnea* larvae (2nd instar) were purchased from a commercial biofactory (Koppert, Berkel en Rodenrijs, The Netherlands). The larvae were used in experiments immediately after delivery.

### 4.3. Bioassays

#### 4.3.1. Acute Toxicity

Wingless adults of *M. dirhodum* (15 per plant pot) were transferred using a fine brush to young wheat plants (grown in universal substrate, pot diameter 9 cm, 5 plants per pot, BBCH 11).

The adults were allowed to move freely along the plants to settle down and begin to suck. The tested substances were turned into a concentration series of aqueous emulsions using the Tween 20 emulsifier (blending ratio 1:10, Tween/compounds) and HG15A homogenizer (5000 revolutions/min, blended for 1 min). At least 5 concentrations were applied in each case, showing mortality rates of 20–90% according to preliminary tests. Specifically: 0.5, 1.0, 1.5, 2.5, and 3.5 mL L^−1^ for carvacrol, 0.25, 0.75, 1.25, 1.75, and 2.25 mL L^−1^ for β-citronellol, 0.5, 1.0, 1.5, 2.5, 3.5, 4.5, and 5.5 mL L^−1^ for isoeugenol, and 2.5, 4.0, 5.0, 6.0, 7.0, and 8.0 mL L^−1^ for linalool. The final concentration of Tween 20 was increased in the application emulsion to always correspond to a concentration of 3.0 mL L^−1^, also achieving a reduction in the water surface tension and even adhesion of the spray liquid. The application was made using a laboratory application sprayer Biostep Sge1 (Desaga-SARSTEDT-Gruppe, Nümbrecht, Germany) at a dose of 5 mL per pot, corresponding approximately to an estimated field application dose of 500 L ha^−1^. The negative control was only treated with water with the respective concentration of Tween 20. To prevent undesirable migration of the tested insects, the plant pots were insulated using breathable insulators after the application. The positive control was the commercial product Neudosan, which was applied in an identical fashion at the concentration recommended by the manufacturer (20 mL L^−1^).

The treated plants were placed in a greenhouse where the temperature was maintained at 21 ± 3 °C, 65 ± 5% R.H., and a 16:8 (L:D) photoperiod. Each treatment was replicated 5 times. Mortality was assessed 48 h after application.

#### 4.3.2. Acute Effects of the Binary Mixtures

The tests were performed using the identical method specified in the Acute Toxicity section, with the concentrations of individual substances or their binary combinations (1:1) corresponding to the estimated LC_30_ (i.e., 0.32, 0.37, 0.45, and 2.50 mL L^−1^ for carvacrol, β-citronellol, isoeugenol, and linalool, respectively). The post-application experiment conditions were identical, as specified above.

The observed mortality values were compared with the expected mortality using the standard formula [26]:

$$E=O_{a}+O_{b}(1-O_{a})$$

where E is the expected mortality caused by the binary mixture, and Oa and Ob are the observed mortalities of the pure substances at concentrations equal to the LC_30_ estimated for *M. dirhodum*.

Whether there is a synergic, antagonist, or no effect between the substances was determined using the formula

$$\chi^{2}=(O_{m}-E{)}^{2}/E$$

where O_m_ is the observed mortality caused by the binary mixture, E is the expected mortality caused by the binary mixture, and $\chi^{2}$ with df = 1 and *p* = 0.05 equals 3.84. A binary mixture for which $\chi^{2}$ > 3.84 and whose observed mortality is higher than the estimated mortality is regarded as synergic. If $\chi^{2}$ < 3.84, there is no mutual effect between the substances [10,26].

For the binary mixture that showed the highest synergic effect in the tests (linalool/ β-citronellol), we subsequently tested various ratios to check that the test ratio 1:1 used is optimal in terms of its effect on aphid mortality. Linalool and β-citronellol were mixed at ratios of 7:1, 1:1, 1:2, and 1:5. Afterwards, emulsions were made for each mixture at concentrations of 0.5, 1.5, and 2.5 mL L^−1^. The experiment design method and post-application conditions were identical, as specified in the Acute Toxicity section.

#### 4.3.3. Inhibition of Fertility and Potential Natality of *Metopolophium dirhodum*

To determine the effect of linalool and β-citronellol, including their binary mixture (1:1), on the fertility of females of *M. dirhodum*, we employed the same method as specified in the Acute Toxicity section to apply emulsions at concentrations corresponding to the estimated LC_30_ and LC_50_ (i.e., 0.37 and 0.59 mL L^−1^ for β-citronellol, 2.50 and 3.68 mL L^−1^ for linalool, and 0.36 and 0.56 mL L^−1^ for their binary mixture). Forty-eight hours after the application, the females were transferred to new young wheat plants, and then the numbers of newborn nymphs were recorded for a period of 7 days. Newborn nymphs were removed with a brush after they had been counted so as to avoid multiple counting.

The experiment was performed at a temperature of 21 ± 3 °C, 65 ± 5% R.H., and a 16:8 (L:D) photoperiod. The experiment was repeated 5 times.

Fertility was expressed as the number of newly hatched nymphs per surviving treated female per day. Fertility inhibition then expresses the percentage by which the number of laid nymphs was reduced compared to the control. Potential birth rates are then expressed by the number of hatched nymphs that a population of 100 treated females will produce in one day, assuming that their 30% or 50% mortality occurs within 24 h (for females treated with LC_30_ or LC_50_, respectively). Potential natality was calculated according to the following formula: Nat = average number of nymphs laid by 100 treated females * predicted mortality coefficient, where the mortality coefficient = 0.7 for aphids treated with concentrations corresponding to the estimated LC_30_, or 0.5 for aphids treated with concentrations corresponding to the estimated LC_50_ [48].

#### 4.3.4. Acute Toxicity against *Aphidoletes aphidimyza* and *Chrysoperla carnea*

Larvae of *A. aphidimyza* in the 3rd instar and larvae of *C. carnea* in the 2nd instar were treated with linalool, β-citronellol, and their binary mixture at concentrations corresponding to the estimated LC_90_ for *M. dirhodum* (i.e., 9.46, 1.78, and 1.58 mL L^−1^). The larvae were dipped for 3 seconds into the test solutions (tested substance + 3 mL L^−1^ of Tween 20) and then put in plastic cups lined with filter paper and covered with breathable caps. Each iteration used 15 larvae. Larvae of *C. carnea* were kept individually to avoid potential cannibalism. Their food consisted of aphids administered to the larvae ad libitum.

The experiment was maintained at a temperature of 21 ± 3 °C, 65 ± 5% R.H., and a 16:8 (L:D) photoperiod. The experiment was repeated 5 times. Mortality was assessed 48 h after application.

### 4.4. Data Analysis

*Metopolophium dirhodum* mortality rates observed in acute toxicity experiments were adjusted according to Abbott [49]; then, LC_50_ and LC_90_ with 95% confidence interval (Cl95) were estimated through probit analysis [50].

Percentage data on the inhibition of aphid fertility and potential natality, as well as mortality data of *A. aphidimyza* and *C. carnea*, were transformed through arcsine square root transformation before being analyzed by ANOVA, followed by Tukey’s HSD test (*p* ≤ 0.05). For all statistical analyses, Biostat 5.9.8 was the software used.

## Figures and Tables

**Table 1:** Estimated LC_30,50,90_ of β-citronellol, carvacrol, isoeugeol, and linalool for *Metopololphium dirhodum* adults.

	LC30 * (CI95)	LC50 * (CI95)	LC90 * (CI95)	χ^2^ (df = 4)	*p*-Value
β-Citronellol	0.37 (0.29–0.44)	0.59 (0.49–0.68)	1.78 (1.50–2.21)	2.990	0.393 ns
Carvacrol	0.32 (0.19–0.44)	0.60 (0.43–0.75)	2.82 (2.23–4.08)	1.918	0.590 ns
Isoeugenol	0.45 (0.27–0.63)	1.09 (0.82–1.35)	9.50 (6.64–16.62)	1.574	0.904 ns
Linalool	2.50 (1.97–2.92)	3.68 (3.20–4.07)	9.46 (8.10–11.99)	3.716	0.446 ns

Neudosan (active ingredient: potassium salts of fatty acids), negative control = Tween 20, 0.3% solution in water; * Concentration—LC_30(50,90)_ in mL L^−1^ causing 30, (50, 90%) mortality of aphids 48 h after application. CI95—95% confidence intervals, activities of extract and compounds are considered significantly different when the 95% CI fails to overlap. Chi-square value, not significant (ns).

**Table 2:** Effect of binary mixtures of compounds on mortality against *M. dirhodum* larvae.

Compound A	Compound B	Larval Mortality (%)				χ^2^	Effect
		Pure Compounds		Binary Mixtures			
		Observed A *	Observed B *	Expected	Observed		
Linalool	Isoeugenol	11.8	22.0	31.2	38.2	1.569	No effect
Linalool	Carvacrol	11.8	11.8	22.2	48.5	31.129	Synergistic
Linalool	β-Citronellol	11.8	32.4	40.4	83.8	46.699	Synergistic
Isoeugenol	Carvacrol	22.0	11.8	31.2	52.9	15.085	Synergistic
Isoeugenol	β-Citronellol	22.0	32.4	47.2	44.2	0.200	No effect
β-Citronellol	Carvacrol	32.4	11.8	40.4	38.2	0.117	No effect

All substances are in concentrations equal to LC_30_ estimated on *Metapolophium dirhodum*; * mortality was corrected using Abbott.

**Table 3:** Acute toxicity of linalool/β-citronellol binary mixtures in 4 different ratios against *Metopololphium dirhodum* adults.

Linalool/β-Citronellol Ratio	Concentrations of Binary Mixtures (mL L^−1^)		
	0.5	1.5	2.5
	Mortality (% ± SD)		
7:1	12.7 ± 2.3 bc	17.1 ± 5.1 c	62.1 ± 7.1 c
1:1	23.4 ± 3.4 a	83.5 ± 2.3 a	97.8 ± 3.2 a
1:2	16.1 ± 3.5 ab	75.2 ± 6.9 ab	80.2 ± 3.4 b
1:5	4.1 ± 4.9 c	58.8 ± 12.9 bc	80.1 ± 3.9 b
ANOVA F3,8; P	9.375; 0.005	27.636; 0.000	11.732; 0.002

Within a column, different letters indicate significant differences among means (ANOVA, Tukey’s HSD test, *p* < 0.05).

**Table 4:** Acute toxicity of binary mixture linalool/β-citronellol (1:1 ratio) against *Metopololphium dirhodum* adults.

Concentration of Linalool and β-Citronellol (mL L^−1^)	Mortality * (% ± SD)	LC_30_ ** (CI_95_)	LC_50_ ** (CI_95_)	LC_90_ ** (CI_95_)	χ^2^ (df = 4)	*p*-Value
0.25	15.9 ± 11.0	0.36 (0.30–0.43)	0.56 (0.48–0.63)	1.58 (1.37–1.89)	4.226	0.376 ns
0.5	27.5 ± 14.5					
1.0	44.9 ± 17.5					
1.25	81.1 ± 17.5					
1.5	91.3 ± 9.4					
2.0	94.2 ± 6.1					
2.5	97.1 ± 4.0					
Negative control	8.0 ± 8.7					
Positive control	100.0 ± 0.0					

* Mortality was corrected using Abbott; positive control = 20 mL L^−1^ Neudosan (active ingredient: potassium salts of fatty acids), negative control = Tween 20, 0.3% solution in water; ** Concentration—LC_30(50,90)_ in mL L^−1^ causing 30, (50, 90%) mortality of aphids 48 h after application. CI95—95% confidence intervals, activities of extract and compounds are considered significantly different when the 95% CI fails to overlap. Chi-square value, not significant (ns).

**Table 5:** Sublethal effect of β-citronellol, linalool, and their binary mixture on fertility and potential natality of *Metapolophium dirhodum* aphids.

	Fertility		Potential Natality	
Treatment	No. Nymphs/Female/Day	Inhibition (%) Compared to Control	No. Nymphs per 100 Females/Day	Inhibition (%) Compared to Control
β-Citronellol: LC_30_	1.60 ± 0.45 b	45.94 ± 15.25 a	112.21 ± 31.61 c	62.16 ± 10.68 a
β-Citronellol: LC_50_	1.94 ± 0.29 b	34.45 ± 12.16 a	96.99 ± 13.69 c	67.22 ± 11.97 a
Linalool: LC_30_	2.74 ± 0.65 a	7.43 ± 22.26 b	191.81 ± 46,13 b	35.20 ± 15.58 b
Linalool: LC_50_	2.68 ± 0.44 a	9.45 ± 15.01 b	134.03 ± 22.22 c	54.72 ± 7.51 a
β-Citronellol/Linalool LC_30_	2.24 ± 0.13 ab	24.32 ± 4.58 a	156,82 ± 9.49 bc	47.02 ± 3.20 ab
β-Citronellol/Linalool LC_50_	2.01 ± 0.12 b	32.43 ± 4.27 a	99.98 ± 6.32 c	66.21 ± 2.13 a
Negative control	2.96 ± 0.46 a		296.05 ± 46.31 a	
ANOVA F_2,12_; P	6,28; 5.924; 0.000	5, 24; 6.176; 0.000	6, 28; 22.841; 0.000	5, 24; 5.167; 0.002

Within a column, different letters indicate significant differences among means (ANOVA, Tukey’s HSD test, *p* < 0.05).

**Table 6:** Acute toxicity of β-citronellol, linalool, and their binary mixture (1:1 ratio) LC estimated on *Metapolophium dirhodum* against *A. aphidimyza*.

*A. aphidimyza* Mortality (% ± SD)					
Treatment	β-Citronellol	Linalool	β-Citronellol/Linalool	Positive Control	Negative Control
Aphid LC_90_	0.0 ± 0.0	3.3 ± 3.8	1.7 ± 3.3	3.3 ± 3.3	1.7 ± 3.3
ANOVA F_4_, _10_; P	ns				

No significant differences among means (ANOVA, Tukey’s HSD test, *p* < 0.05). Negative control = Tween 20, 0.3% solution in water; positive control = 20 mL L^−1^ Neudosan (active ingredient: potassium salts of fatty acids).

**Table 7:** Acute toxicity of β-citronellol, linalool, and their binary mixture (1:1 ratio) LC estimated on *Metapolophium dirhodum* against *C. carnea*.

*C. carnea* Mortality (% ± SD)					
Treatment	β-Citronellol	Linalool	β-Citronellol/Linalool	Positive Control	Negative Control
Aphid LC_90_	35.3 ± 9.9 b	4.8 ± 8.2 c	19.6 ± 4.7 cb	100.0 ± 0.0 a	9.0 ± 3.7 c
ANOVA F_4_, _10_; P	112.915; 0.000				

Within a column, different letters indicate significant differences among means (ANOVA, Tukey’s HSD test, *p* < 0.05). Negative control = Tween 20, 0.3% solution in water; positive control = 20 mL L^−1^ Neudosan (active ingredient: potassium salts of fatty acids).

## Data Availability

The data presented in this study are available on request from the corresponding authors.
